# Fasting ghrelin as mediator between obesity and depressive symptoms: a pre-registered study

**DOI:** 10.1038/s44184-026-00217-2

**Published:** 2026-05-19

**Authors:** Konrad Jakob Endres, Laurenz Lammer, Frauke Beyer, Daria E. A. Jensen, Dirk Alexander Wittekind, Margrit Löbner, Arno Villringer, Julia Sacher, A. Veronica Witte

**Affiliations:** 1 Department of Neurology, Max Planck Institute for Human Cognitive and Brain Sciences, Leipzig, Germany; 2 Cognitive Neurology, University of Leipzig Medical Center, Leipzig, Germany; 3 Bordeaux Population Health Research Center, Inserm, University of Bordeaux, Bordeaux, France; 4 LeiCeM - Leipzig Center of Metabolism, Leipzig University, Leipzig, Germany; 5 Institute of Laboratory Medicine, Clinical Chemistry and Molecular Diagnostics, Leipzig University, Leipzig, Germany; 6 Department of Psychiatry and Psychotherapy, Leipzig University, Leipzig, Germany; 7 Institute of Social Medicine, Occupational Health and Public Health, Faculty of Medicine, Leipzig University, Leipzig, Germany; 8 Center for Integrative Female Health & Gender Medicine, University of Leipzig Medical Center, Leipzig, Germany; 9 Medical Department III – Endocrinology, Nephrology, Rheumatology, University Leipzig, Leipzig, Germany; 10 German Center for Child and Adolescent Health (DZKJ), partner site Leipzig/Dresden, Greifswald, Germany

**Keywords:** Obesity, Neuroscience, Biomarkers, Epidemiology, Translational research

## Abstract

Ghrelin, a hunger-related gut hormone, may contribute to higher risk of depressive symptoms in obesity. Despite animal studies suggesting antidepressant effects of circulating ghrelin, human studies remain inconclusive. Therefore, we aimed to explore the association between obesity, ghrelin serum levels, and depressive symptoms in a large population-based cohort. Assessments of the LIFE-Adult cohort (*n* = 6037, 18–82 years) included questionnaires to evaluate depressive symptoms (CES-D and IDS-SR), anthropometric measurements for BMI, fasting ghrelin serum levels via radioimmunoassay (*n* = 1089), and 3 T MRI for hippocampal volume (*n* = 1080). Statistical analyses were pre-registered (https://osf.io/y7sbx). Higher BMI predicted more frequent depressive symptoms (*β* = 2.033, *p* < 0.001) and lower fasting serum ghrelin (*β* = −0.622, *p* < 0.001). Ghrelin did not correlate with depressive symptoms in obesity (*n* = 263, *β* = 0.123, *p* = 0.918). Exploratory analyses revealed links between ghrelin and eating-related depressive symptoms, and that higher BMI was more strongly associated with depressive symptoms in females than males. In this large, well-characterized sample, ghrelin was not associated with overall severity of depressive symptoms in participants with obesity. Future studies using more specific ghrelin assessments and clinical samples could help clarify this relationship.

## Introduction

Obesity has become a major global health challenge. According to the World Health Organization in 2022, 1 in 8 people in the world were living with obesity. The worldwide prevalence of obesity nearly tripled between 1975 and 2016^1^. A high body mass index (BMI) is a risk factor for various diseases, including neurodegenerative and cardiovascular disorders, type 2 diabetes, chronic kidney disease, and cancer^2^. Additionally, obesity has a significant impact on mental health and was associated with an increase in the odds ratio of mood disorders by approximately 25%^3^. A large meta-analysis and systematic review of nearly 2 million participants from 29 cross-sectional, 6 cohort, and 8 randomized controlled studies suggests that obesity increases the risk of depression^4^.

Obesity and depression are linked in a bidirectional and multifactorial manner. Weight-related stigma and low-grade chronic inflammation are commonly described to contribute to this relationship^5^. In addition, the increased susceptibility of individuals living with obesity to depressive symptoms could be due to changes in their endocrine system. For instance, human studies found that obesity correlates with lower circulating levels of ghrelin^6–8^, which might increase the risk of developing depressive symptoms^9^.

Ghrelin is a short orexigenic peptide produced by enteroendocrine cells of the stomach^10^. Under physiological conditions, ghrelin activates arcuate nucleus neurons in the hypothalamus, inducing hunger and counteracting satiety signals like leptin. Ghrelin reaches the hypothalamus through the circumventricular organs^11^. Alternatively, ghrelin can reach the brain via active transport across the blood-brain-barrier^12^. Beyond the hypothalamus, ghrelin affects brain regions linked to depressive symptoms, including the hippocampus, amygdala, pituitary gland, and ventral tegmental area^13,14^.

Specifically, ghrelin has been suggested to exert antidepressant effects based on evidence from animal studies^15^. For example, in the forced swim test, mice with elevated ghrelin levels had a longer latency to immobility and a shorter time of total immobility, indicating less depressive-like symptoms^9^. In that same study, mice with a genetic blockade of ghrelin expression exhibited more signs of social avoidance after chronic stress compared to their wild-type littermates with elevated ghrelin^9^. This may suggest a protective role of ghrelin against maladaptive stress responses, noting that reduced social interest is a common symptom of depression. Moreover, ghrelin administration led to less depressive-like symptoms and increased memory retention in rats^16^.

Human studies investigating whether depressive symptoms or psychiatric disorders are linked to a disbalance in ghrelin levels offered inconclusive results. Some studies showed lowered ghrelin levels in patients with major depressive disorder (MDD) compared to healthy controls^17,18^ while other studies found higher levels^19–21^, or no significant difference^22–25^. Similarly, ghrelin did not correlate with self-rating depression scores in a previous analysis of a population-based sample^26^. However, some of the included patients in those studies received medication or electroconvulsive therapy, rendering definite conclusions difficult. While electro-convulsive therapy decreased ghrelin levels in patients with mood disorders^20^, medication was shown to normalize ghrelin levels in patients with increased ghrelin serum levels^19^. None of the studies included populations with a mean BMI ≥ 30 kg/m², despite the need to examine ghrelin–depression associations in individuals with obesity, who show reduced ghrelin levels^27^ and higher prevalence of depressive symptoms (29), suggesting obesity-specific mechanisms.

In major depression, a single-blind, placebo-controlled study found no significant change in self-rated symptoms after 4 × 50 µg ghrelin, though a trend toward improvement was noted in the small sample of seven male participants^28^. Given that females are more frequently affected by depression^29^ and have higher levels of ghrelin expression^30^, sex is an important variable that could influence the outcomes of ghrelin’s antidepressant effects. Interestingly, ghrelin was found to stimulate estrogen secretion, creating a positive feedback loop that may enhance its antidepressant potential^31^.

Several mechanisms could underlie the putative antidepressant effects of ghrelin. Firstly, studies suggest that ghrelin increases serotonergic transmission in the brain through increasing mRNA expression of serotonin-related genes^32–34^. Enhanced serotonergic neurotransmission has been discussed to mediate better mood and sleep in human patients with depression, for example after the intake of selective serotonin-reuptake inhibitors (SSRIs)^34,35^, however see ref. ^36^ for recent discussions. Furthermore, higher endogenous ghrelin after caloric restriction stimulated neurogenesis in the hippocampus of mice^37^, an area that has been implicated in the neurobiology of depressive symptoms^38^. Another key mechanism of ghrelin in the hippocampus involves cAMP-mediated activation of the cAMP response-element binding protein, which drives synaptic plasticity and is modulated by endocannabinoids, collectively shaping hippocampal appetitive learning and memory processes^39^. The hippocampus serves as a critical region of interest in this study for two key reasons: once the aforementioned ghrelin-mediated influence on hippocampal neurogenesis and synaptic plasticity, and the established role of the hippocampus in the pathophysiology of major depression^40^, highlighting its relevance to obesity-depression comorbidity. Notably, individuals with major depressive disorder demonstrated reduced hippocampal volumes relative to healthy controls^41^.

In sum, animal studies suggest that ghrelin may exert antidepressant effects via neuroprotection in the hippocampus, and obesity-related reductions in ghrelin levels^42,43^ could partly explain the higher susceptibility to depressive symptoms in individuals with obesity. However, human evidence on antidepressant effects of ghrelin and its link to hippocampal integrity is scarce, and it remains unclear to what extent fasting ghrelin mediates the BMI–depressive symptoms association. In this study we thus aim to systematically address this issue by analyzing the relationship between BMI, fasting ghrelin serum levels and depressive symptoms in a large population-based cohort including individuals living with obesity and assessments of ghrelin levels, depressive symptoms and structural neuroimaging.

To this end we have tested the following pre-registered hypotheses (https://osf.io/y7sbx):

**H1)** A higher BMI is associated with more frequent depressive symptoms and

**H2)** with lower fasting ghrelin serum levels in an adult population without major acute disease.

**H3)** Lower fasting ghrelin serum levels are associated with more frequent depressive symptoms in individuals living with obesity.

**H4)** The association between BMI and depressive symptoms is mediated by lowered fasting ghrelin levels.

To confirm that obesity (compared to normal weight) relates to more frequent depressive symptoms (H1) and that obesity (compared to normal weight) often goes in line with lower ghrelin levels (H2), we investigated these measures in relation to the full BMI spectrum. While our first two hypotheses primarily serve to confirm well-established findings, our study adds to the literature by focusing on the LIFE-Adult cohort, which includes a middle- to old-aged population with increasing BMI over time, a group that has not been extensively studied in relation to depressive symptoms and metabolic changes associated with obesity as a potential link to mental health outcomes^44^. Furthermore, our population-based sample is drawn from a rural and urban region in Eastern Germany, a setting characterized by unique socioeconomic conditions and a distinct prevalence of obesity, which may influence the relationship between obesity and depressive symptoms^45^.

Studies examining the association between fasting ghrelin serum levels and hippocampal volume as a marker of hippocampus integrity in a population-based sample of humans are still missing. Given the pro-neurogenic properties of ghrelin in mice, we hypothesized that ghrelin levels are positively associated to hippocampal volume.

## Methods

### Study design and subjects

We used data from the LIFE-Adult population-based cohort study which randomly selected 10,000 participants from Leipzig^44,46^. The baseline examination was completed in November 2014 and included questionnaires, anthropometry, blood measurements, and MRI. The blood measurements were performed after an overnight fast for standardization.

**Fig. 1:**
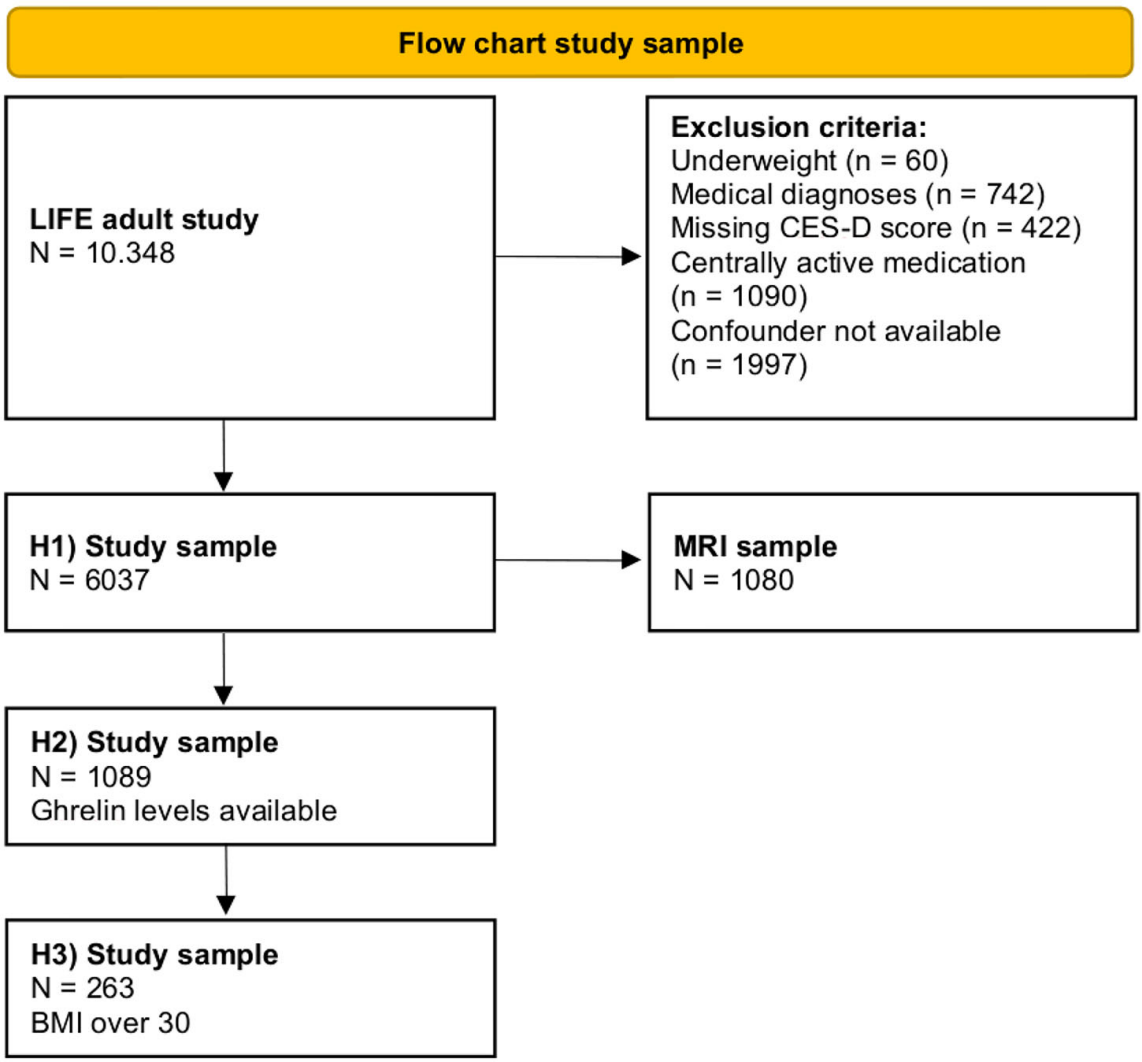
Flow chart with exclusion criteria and sample sizes.

We excluded participants with acute severe disease (except MDD, diabetes mellitus type 2, obesity, hyperlipidemia, or hypertension). To avoid confounding by antidepressant treatment, we excluded participants with psychotropic medication. Additionally, we excluded all participants with a BMI lower than 18.5 kg/m² which is considered to be underweight according to the WHO and participants with missing or invalid Center for Epidemiological Studies Depression Scale (CES-D) scores. Underweight individuals exhibit elevated ghrelin levels^47^, which could confound the effects of BMI and obesity on ghrelin levels in a linear regression model. Furthermore, underweight is associated with psychiatric conditions such as anorexia nervosa^48^, which could confound the association between BMI and depressive symptoms. Due to the exclusion criteria, the sample size was reduced from 10,348 in the original LIFE-Adult data set to 6037 in our study sample for the first hypothesis. Out of the 6037 participants eligible for our analyses, 1089 had ghrelin measurements (see Fig. 1).

Written informed consent was obtained from all participants who agreed to participate in the study. The study procedures were carried out in compliance with the ethical principles of the Declaration of Helsinki and were approved by the Ethics Committee of Leipzig University (registration number: 263-2009-14122009).

The project was preregistered with hypotheses, analysis plan, and statistical models on the open science framework (https://osf.io/y7sbx).

### Assessment of body mass index (BMI)

In order to calculate the BMI, the body weight and height of the participants were measured. The body weight measurement was performed by an electronic scale (SECA 701, SECA Gmbh & Co KG) with an inaccuracy of ±0.01 kg. The height was determined by a trained professional with a stadiometer (SECA 204) with an inaccuracy of ±0.1 cm following a standard operating procedure.

### Assessment of depressive symptoms

We evaluated depressive symptoms with two questionnaires: the Inventory of Depressive Symptoms – Self Rating *IDS-SR* and the Center for Epidemiological Studies Depression Scale *CES-D*). Both questionnaires measure self-rated depressive symptoms. The CES-D measures self-reported depressive symptoms over the past week with 20 items. Each item can be rated from 0 to 3 with a Likert-scale leading to the highest possible sum score of 60^49^. Even though a cut-off score of ≥20 reflects severe depressive symptoms and may suggest the presence of a depressive episode, it does not constitute a diagnostic criterion for major depression^50^. The IDS-SR has 30 items that can be scored from 0 to 3 as well^51^. Higher sum scores in the questionnaires (IDS-SR or CES-D) indicate more depressive symptoms.

We used the CES-D for confirmatory analysis because of fewer missing items. However, we used the IDS-SR for the exploratory analysis, as it distinguishes between losing and gaining appetite as a depressive symptom.

### Ghrelin serum level measurement

Ghrelin levels were measured in blood serum collected after an overnight fast via a radioimmunoassay (Mediagnost, Reutlingen Germany). As only total ghrelin levels were measured, we could not distinguish between acyl- and desacyl-ghrelin. Blood samples were not treated with enzyme inhibitors like aprotinin. The assay had a sensitivity of 0.04 ng/mL and a mean intra-assay coefficient of variation between 6.9 and 9.2%.

### MRI

T1-weighted images were acquired using a 3 Tesla Siemens Verio MRI scanner (Siemens Healthcare, Erlangen, Germany) with a 3D MPRAGE protocol. The MRI parameters included an inversion time of 900 ms, a repetition time of 2300 ms, an echo time of 2.98 ms, a flip angle of 9°, a field of view of 256 × 240 × 176 mm^3^, and a voxel size of 1 × 1 × 1 mm^3^. The shimming was performed using a tune-up shim, with no fat suppression, and the imaging covered the entire brain. We utilized FreeSurfer (version 5.3.0, RRID:SCR_001847) to analyze the scans using the standard cross-sectional pipeline recon-all. FreeSurfer performed automated measurements of hippocampal volume. For further details on the preprocessing and quality control see ref. ^52^.

### Statistical analyses

We used hierarchical linear regression models in R to test H1–3. We had preregistered to conduct the mediation analysis using the method proposed by Baron and Kenny to test H4. However, we were unable to apply this method because the prerequisites for mediation analysis were not met. Specifically, H3 resulted in a null finding, which prevented us from proceeding with the analysis as initially planned.

The assumptions of linear regression models (e.g., normality, homoscedasticity and absence of mulitcollinearity) were checked visually using histograms of the predictor variables and plotting fitted value vs. residual plots. We performed either asinh- or log-transformation in case of skewed distributions of the variables.

The statistical significance of the effects was assessed with full-null-model comparisons and p-values were Bonferroni-adjusted for multiplicity control. We performed a likelihood-ratio test to assess the difference between the null hypotheses and alternative hypotheses. Furthermore, we reported the according β-estimates and p-values. The significance level was predefined as *α* = 0.05 and after Bonferroni-correction *α* = 0.01666 (*α* /*n* = 0.05/3).

We controlled our linear regression analyses for several confounders that had been shown to affect ghrelin serum levels, depressive symptoms, or BMI. To this end we included age, self-reported sex, smoking status, alcohol consumption, sleep, season, undiagnosed thyroid disease, and diabetes as control variables. We introduced the control variables step by step into the model to test if they improved the fit of the model. To this end, we created various models that gradually included more control variables. With the likelihood-ratio test, we tested if the more complex model had significantly better fits.

As ghrelin was shown to improve hippocampal neurogenesis in mice^37^ we analyzed the association between ghrelin serum levels and hippocampal volume. Additionally, we calculated Bayes factors for the third hypothesis to provide a quantitative measure of the strength of evidence for the absence of an effect. To explore sex differences in H1–3 and exploratory hypotheses regarding the association between ghrelin levels and hippocampal volume, we calculated the interaction term between sex and the predictor variable outlined in each hypothesis.

R-Syntax for the statistical models:

- Model_1:
  - Model_1.0 = lm(CESD ~ 1)
  - Model_1.1 = lm(CES-D ~ BMI)
  - Model_1.2 = lm(CES-D ~ BMI + age + sex)
  - Model_1.3 = lm(CES-D ~ BMI + age + sex + smoking + alcohol_consumption + physical_activity + thyroid_hormones + season + diabetes)
- Model_2:
  - Model_2.0 = lm(serum_ghrelin ~ 1)
  - Model_2.1 = lm(serum_ghrelin ~ BMI)
  - Model_2.2 = lm(serum_ghrelin ~ BMI + age + sex)
  - Model_2.3 = lm(serum_ghrelin ~ BMI + age + sex + smoking + alcohol_consumption + sleep + physical_activity + thyroid_hormones + season + diabetes)
- Model_3:
  - Model_3.0 = lm(CES-D ~ 1)
  - Model_3.1 = lm(CES-D ~ serum_ghrelin)
  - Model_3.2 = lm(CES-D ~ serum_ghrelin + age + sex)
  - Model_3.3 = lm(CES-D ~ serum_ghrelin + age + sex + smoking + alcohol_consumption + physical_activity + thyroid_hormones + season + diabetes)
- Exploratory models:
  - Model_4.3 = lm(HCV_left ~ serum_ghrelin + age + sex + smoking + alcohol_consumption + sleep + physical_activity + thyroid_hormones + season + diabetes + ICV)
  - Model_5.3 = lm(HCV_right~ serum_ghrelin + age + sex + smoking + alcohol_consumption + sleep + physical_activity + thyroid_hormones + season + diabetes + ICV)

### Confounder variables

#### Smoking status

Smoking status was evaluated with a structured interview, separating groups of active smokers, former smokers and non-smokers for comparison^53^.

#### Alcohol consumption

A food frequency and alcohol questionnaire (FFQ) was administered to the participants^54^. We controlled for alcohol consumption as a continuous variable with the pure alcohol intake in g/day. The average alcohol intake was calculated by the frequency of alcohol consumption, amount and the alcohol content of the kind of beverage.

#### Physical activity

Physical activity was assessed with the International Physical Activity Questionnaire (IPAQ)^55^. With the information on frequency, duration and intensity of physical activity MET-minutes per week were calculated. We used MET-minutes per week to control for physical activity as a continuous variable.

#### Sleep

We used the self-reported Pittsburgh Sleep Quality Index that assesses the sleep quality and sleep disturbance of the participants and calculated the PSQI sum score according to the guidelines^56^. The PSQI sum score ranges from 0 to 21, with higher scores indicating worse sleep quality.

#### Diabetes

We controlled for diabetes using a dichotomous variable. Participants were considered diabetic if they had either had a diagnosis, a HbA1c over 6.5% or were taking antidiabetic medication. We did not differentiate diabetes types.

#### Season

We controlled for seasonal differences in BMI and depressive symptoms^57^ by creating a new variable differentiating participants that had their assessment in spring or summer and those assessed in fall or winter.

#### Thyroid hormones

We excluded all participants with a diagnosis of thyroid disease from due to its impact on BMI and depressive symptoms^58^. Nonetheless, we controlled for undiagnosed thyroid diseases by creating a group of hypothyroid, euthyroid and hyperthyroid participants. Participants with either high TSH, low FT3, or low FT4 were considered hypothyroid. Participants with either low TSH, high FT3 or high FT4 were considered hyperthyroid. Participants without alterations in the serum levels of the hormones TSH, FT3, and FT4 were considered euthyroid. Thyroid function parameters (TSH, FT3, FT4) were assessed using electrochemiluminescence assays (ECLIA) performed on Cobas 601 or 801 instruments from Roche Diagnostics, Germany.

#### Total intracranial volume (ICV)

In the exploratory analyses examining hippocampal volume, total intracranial volume was included as a covariate to account for potential confounding effects of interindividual differences in overall brain size.

### Exploratory analysis

In further analyses we explored whether ghrelin levels correlated with specific physical depressive symptoms, such as changes in appetite and weight, psychomotor symptoms (psychomotor retardation, restlessness, leaden paralysis, energy level), changes in sleep and gastrointestinal symptoms, using linear regression models.

We therefore analyzed following sub-items of the IDS-SR questionnaire:

- sumscore sleep disturbances (problems falling asleep, sleep during the night, waking up too early, and hypersomnia): IDS items 1,2, 3, and 4;
- decreased appetite: IDS item 11;
- increased appetite: IDS item 12;
- decrease of weight: IDS item 13;
- increase of weight: IDS item 14;
- sumscore psychomotor symptoms: IDS items 20, 23, 24, and 30;
- constipation/diarrhea (gastrointestinal symptoms): IDS item 28

## Results

### Study sample characteristics

Differences in sample size for the different hypotheses emerged due to discrepancies in the availability of variables of interest. The initial sample was population-based and limited to participants with available CES-D (H1). The sample for H2) and H3) based on additional blood measures that were available in a subset only. Additionally, in the sample for H3), only individuals with a BMI categorized as obese were included and some exploratory analyses based on availability of MRI measures.

This resulted in a higher proportion of women in the sample for H1 (52.39%) compared to the other samples (44.17% for H2, 45.63% for H3 and 44.17% for the MRI sample, see Table 1 for details). Also, the samples differed notably in age, percentage of participants with a depressive episode (CES-D sum score ≥ 20), and smoking status.

**Table 1:** Study sample characteristics

Variables	Sample H1	Sample H2	Sample H3	MRI sample
Sample size (*n*)	6037	1089	263	1080
Age, years (mean ± SD)	59.56 ± 12.28	56.02 ± 15.26	61.76 ± 11.61	55.99 ± 15.30
Sex (n female, %)	3163 (52.39%)	481 (44.17%)	120 (45.63%)	477 (44.17%)
BMI, kg/m² (mean ± SD)	26.96 ± 4.72	26.91 ± 4.41	32.40 ± 3.42	26.91 ± 4.41
Ghrelin serum levels, pg/ml, log-transformed (mean ± SD)	not included	6.76 ± 0.40	6.60 ± 0.32	6.75 ± 0.40
Depressive symptoms (CES-D), sum score (mean ± SD)	9.74 ± 6.18	9.00 ± 5.76	9.87 ± 5.74	8.99 ± 5.76
Clinically depressed participants, CESD sum score ≥20 (*n*,%)	425 (7.04%)	54 (4.96%)	15 (5.70%)	53 (4.91%)
Smoking status				
Non-smoker (*n*,%)	3038 (50.32%)	621 (57.02%)	130 (49.43%)	616 (57.04%)
Former smoker (*n*,%)	1735 (28.74%)	316 (29.02%)	103 (39.12%)	314 (29.07%)
Active smoker (*n*,%)	1264 (20.94%)	152 (13.96%)	30 (11.41%)	150 (12.89%)
Alcohol consumption, g/day, asinh-transformed (mean ± SD)	2.34 ± 1.47	2.34 ± 1.44	2.17 ± 1.56	2.33 ± 1.43
Sleep (PSQI), sum score, asinh-transformed (mean ± SD)	not included	2.25 ± 0.56	not included	2.24 ± 0.55
Physical activity (IPAQ), MET-minutes per week, asinh-transformed (mean ± SD)	8.74 ± 1.16	8.84 ± 0.98	8.84 ± 1.20	8.84 ± 0.98
Diabetes mellitus (*n*, %)	542 (8.98%)	110 (10.10%)	62 (23.57%)	109 (10.09%)
Undiagnosed thyroid disease (*n*,%)	619 (10.25%)	118 (10.84%)	35 (13.31%)	117 (10.83%)
Undiagnosed hypothyroidism (*n*,%)	280 (4.64%)	47 (4.32%)	11 (4.18%)	47 (4.36%)
Undiagnosed hyperthyroidism (*n*,%)	339 (5.62%)	71 (6.52%)	24 (9.13%)	70 (6.48%)
Test days in spring or summer (*n*,%)	3291 (54.51%)	589 (54.09%)	139 (52.85%)	584 (54.07%)
Hippocampal volume				
Left HCV, mm³ (mean ± SD)	not included	not included	not included	3823 ± 481.74
Right HCV, mm³ (mean ± SD)	not included	not included	not included	4017 ± 479.55
Total intracranial volume, mm³ (mean ± SD)	not included	not included	not included	489638 ± 154624.8

*BMI* body mass index, *CES-D* Center for Epidemiological Depression Scale,*HCV* hippocampal volume,*IPAQ* International Physical Activity Questionnaire, *MET* metabolic equivalent of task, *MRI* magnetic resonance imaging, *PSQI* Pittsburgh Sleep Quality Index, *SD* standard deviation.

### Association between BMI and depressive symptoms

**Fig. 2:**
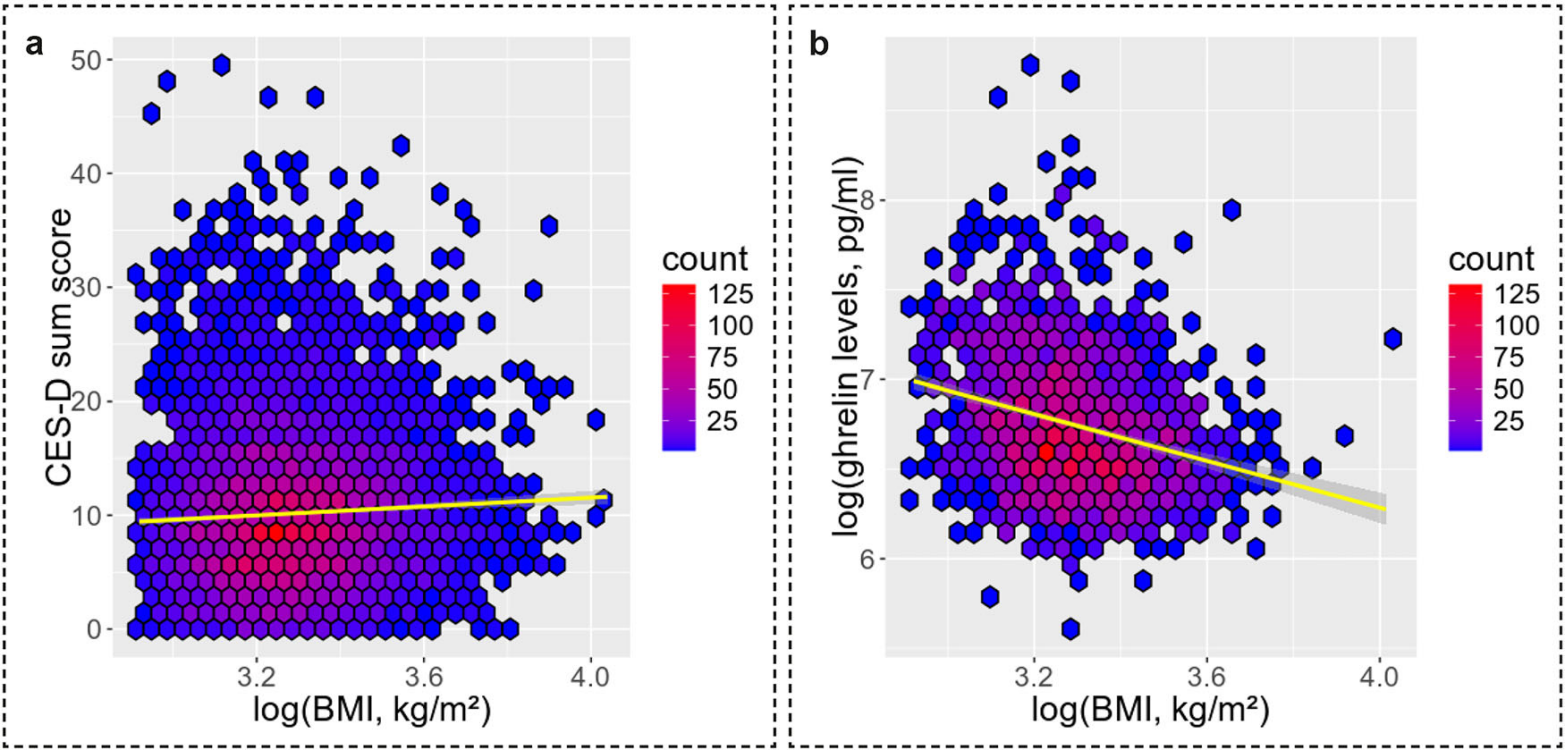
Linear regression models. Outputs of the linear regression models for H1 and H2 depicted in hexbin plots, showing that higher BMI is related to more depressive symptoms (**a**) and lowered ghrelin serum levels (**b**). The color gradient represents the data density, with red for higher and blue for lower data density. Line gives regression fit with 95% confidence intervals. BMI body mass index, CES-D Center for Epidemiological Studies Depression Scale

In the sample for H1 (*n* = 6037) a higher BMI (log-transformed due to non-normal distribution) was associated with more frequent depressive symptoms while controlling for sex, age, smoking status, alcohol consumption, physical activity, diabetes, season and thyroid hormones (Table 2, Fig. 2a, *β*_BMI_ (SE) = 2.033 (0.503), Bonferroni-corrected, *p* < 0.001, adjusted *R*^2^ = 0.026). Results further indicated that depressive symptoms were more frequent in females compared to males. To explore potential sex differences in the relationship between obesity and depressive symptoms, the interaction term between BMI and sex was calculated and added to the model. This revealed a significant effect modification (*β*_BMI*sex_ (SE) = 2.56 (1.045), *p* = 0.014), and stratified analyses suggested that the observed association between higher BMI and more frequent depressive symptoms was primarily driven by females (*ß*_BMI_ (SE), females = 2.99 (0.64), *p* < 0.001 and *ß*_BMI_ (SE), males = 0.43 (0.83), *p* = 0.6).

**Table 2:** Results of multiple linear regression analyses regarding the association between depressive symptoms, BMI and ghrelin levels in the different samples H1, H2, and H3

	*β*	SE	*t*-value	*p*-value	Significance
CES-D sum score in sample for H1 (*n* = 6037) adjusted *R*^2^ = 0.02606, F-statistic = 18.94 on 9 and 6027 DF, *p*-value = <2.2e-16					
Age, years	0.005	0.007	0.709	0.478	
Sex, male/female	1.504	0.174	8.656	<0.001	***
BMI, kg/m², log-transformed	2.033	0.503	4.045	<0.001	***
Smoking Status (active smoker/non-smoker/former smoker)	0.689	0.102			
Alcohol consumption, g/day, asinh-transformed	−0.082	0.059			
Physical activity (IPAQ), MET-minutes per week, asinh-transformed	−0.080	0.068			
Diabetes mellitus, (y/n)	1.082	0.290			
Season, spring and summer vs. fall and winter	−0.110	0.155			
Undiagnosed thyroid disease (euthyroid vs hypothyroid vs hyperthyroid)	0.236	0.246			
Log-transformed Ghrelin serum levels in sample for H2 (*n* = 1089) adjusted *R*^2^ = 0.1519, F-statistic = 20.57 on 10 and 1082 DF, *p*-value = <2.2e-16					
Age, years	<0.001	0.001	−0.061	0.951	
Sex, male/female	0.295	0.025	10.494	<0.001	***
BMI, kg/m², log-transformed	−0.622	0.075	−7.234	<0.001	***
Smoking status (active smoker/non-smoker/former smoker)	0.030	0.015			
Alcohol consumption, g/day, asinh-transformed	0.029	0.008			
Sleep (PSQI), sum score, asinh-transformed	0.015	0.020			
Physical activity (IPAQ), MET-minutes per week, asinh-transformed	−0.005	0.011			
Diabetes mellitus (y/n)	−0.010	0.037			
Season, spring and summer vs. fall and winter	0.032	0.022			
Undiagnosed thyroid disease (euthyroid/hypothyroid/ hyperthyroid)	−0.032	0.033			
CES-D sum score in sample for H3 (*n* = 263) adjusted *R*^2^ = 0.0007418, F-statistic = 1.019 on 10 and 252 DF, *p*-value = 0.4273					
Ghrelin levels, pg/ml, log-transformed	0.123	1.198	0.102	0.918	
Age, years	−0.008	0.033	−0.241	0.809	
Sex, male/female	0.737	0.849	0.868	0.386	
BMI, kg/m², log-transformed	2.627	3.860	0.681	0.497	
Smoking status (active smoker/non-smoker/former smoker)	0.456	0.556			
Alcohol consumption, g/day, asinh-transformed	−0.003	0.020			
Physical activity (IPAQ), MET-minutes per week, asinh-transformed	0.410	0.316			
Diabetes mellitus (y/n)	2.047	0.865			
Season, spring and summer vs. fall and winter	−0.488	0.737			
Undiagnosed thyroid disease (euthyroid/hypothyroid/ hyperthyroid)	1.030	0.994			

Significant codes: *p < 0.017, **p < 0.01, ***p < 0.001.

*BMI* body mass index, *CES-D* Center for Epidemiological Depression Scale, *DF* degrees of freedom, *IPAQ* International Physical Activity Questionnaire, *MET* metabolic equivalent of task, *PSQI* Pittsburgh Sleep Quality Index, *SE* standard error.

### Association between BMI and ghrelin serum levels

In the sample for H2 (*n* = 1089), higher BMI (log-transformed) correlated with lowered fasting ghrelin serum levels (log-transformed) while controlling for aforementioned confounders (Table 2, Fig. 2b, *β*_BMI_ (SE) = -0.622 (0.075), Bonferroni-corrected, *p* < 0.001, adjusted *R*^2^ = 0.152). While sex contributed significantly to the model, adding a BMI*sex interaction term to predict ghrelin levels did not explain further variance (*β*_BMI*sex_ (SE) = 0.231 (0.152), *p* = 0.128).

### Ghrelin serum levels and depressive symptoms in individuals living with obesity

Ghrelin serum levels (log-transformed) did not show a statistically significant association with the CES-D sum score in the sample for H3 (*n* = 263) while controlling for confounders (*β*_ghrelin_ (SE) = 0.123 (1.198), *p* = 0.918, adjusted *R*^2^ <0.001) (Table 2). Similar findings were observed prior to confounder adjustment (*β* (SE) = 0.318 (1.126), *p* = 0.778, adjusted *R*^2 ^< 0.001).

The calculated Bayes factor of 0.56 for the third hypotheses suggests anecdotal evidence in favor of the null hypothesis, indicating a slight tendency towards accepting the null hypothesis. When exploratorily adding an interaction term between ghrelin levels and sex (*β*_ghrelin*sex_ (SE) = 3.97 (2.448), *p* = 0.106) no further variance was explained, indicating no significant sex differences in the association between ghrelin levels and depressive symptoms in this sample of individuals living with obesity.

### Exploratory analyses on ghrelin and specific subdimensions of depressive symptoms

**Fig. 3:**
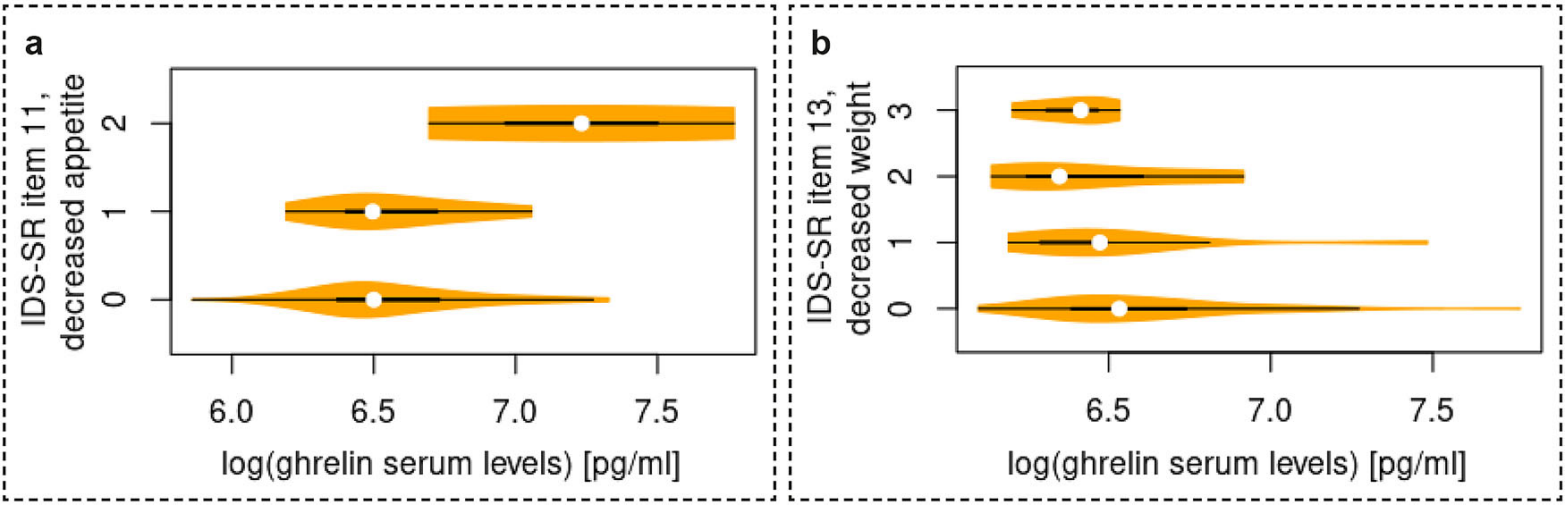
Violin plots regarding the association between ghrelin and specific subdimensions of depressive symptoms. **a** The upper violin-plot illustrates the association between log-transformed ghrelin levels and decreased appetite (IDS-SR item 11) (0 = There is no change in my usual appetite, 1 = I eat somewhat less often or lesser amounts of food than usual, 2 = I eat much less than usual and only with personal effort and 3 = I rarely eat within a 24-h period, and only with extreme personal effort or when others persuade me to eat). **b** The lower violin-plot shows the association between and log-transformed ghrelin levels and decreased weight within the last two weeks (IDS-SR item 13) (0 = I have not had a change in my weight, 1 = I feel as if I’ve had a slight weight loss, 2 = I have lost 2 pounds or more, 3 = I have lost 5 pounds or more). IDS-SR inventory of depressive symptoms–self rating.

Higher log-transformed ghrelin levels were associated with decreased appetite (IDS-SR item 11) (*n* = 263, Fig. 3, *β* (SE) = 0.273 (0.088), *p* = 0.003), and less weight loss within the last two weeks (IDS-SR item 13) (*n* = 263, Fig. 3, *β* (SE) = −0.111 (0.043), *p* = 0.012). No further significant correlations emerged (see SI for details).

### Log-transformed ghrelin levels and hippocampal volume

In exploratory analyses, fasting ghrelin serum levels (log-transformed) were neither associated with left hippocampal volume (β_ghrelin_ (SE) = -4.876 (31.16), p = 0.876) nor with right hippocampal volume (β_ghrelin_ (SE) = 5.312 (34.4), p = 0.877) while controlling for confounders (Table 3).

**Table 3:** Results of multiple linear regression analyses regarding the association between ghrelin levels and right and left hippocampal volume in the MRI sample

	*β*	SE	*t*-value	*p*-value	significance
Right hippocampal volume in MRI sample (*n* = 1080) adjusted *R*^2^ = 0.3054 F-statistic = 37.42 on 13 and 1064 DF, *p*-value = <2.2e-16					
Age, years	−11.280	0.892	−12.652	<0.001	***
Sex, male/female	−22.050	33.370	−0.661	0.509	
Ghrelin levels, pg/ml, log-transformed	5.312	34.400	0.154	0.877	
BMI, kg/m², log-transformed	105.700	87.310	1.211	0.226	
Smoking Status (active smoker/non-smoker/former smoker)	14.990	17.680			
Alcohol consumption, g/day, asinh-transformed	−1.945	0.853			
Sleep (PSQI), sum score, asinh-transformed	−13.000	23.790			
Physical activity (IPAQ), MET-minutes per week, asinh-transformed	−26.670	12.680			
Diabetes mellitus (y/n)	−139.500	42.180			
Season, spring and summer vs. fall and winter	11.510	24.860			
Undiagnosed thyroid disease (euthyroid/hypothyroid/hyperthyroid)	−18.440	37.530			
Depressive symptoms (CES-D), sum score	2.265	2.303			
Total intracranial volume, mm³	0.001	<0.001			
Left hippocampal volume in MRI sample (*n* = 1080) adjusted *R*^2^ = 0.2972 F-statistic = 36.16 on 13 and 1068 DF, *p*-value = <2.2e-16					
Age, years	−11.770	0.8970	−13.117	<0.001	***
Sex, male/female	−10.920	33.24 0	−0.329	0.7425	
Ghrelin levels, pg/ml, log-transformed	−4.876	31.160	−0.156	0.876	
BMI, kg/m², log-transformed	57.650	87.060	0.662	0.508	
Smoking Status (active smoker/non-smoker/former smoker)	−7.781	17.85			
Alcohol consumption, g/day, asinh-transformed	−1.671	0.861			
Sleep (PSQI), sum score, asinh-transformed	−25.170	23.96			
Physical activity (IPAQ), MET-minutes per week, asinh-transformed	−17.620	12.760			
Diabetes mellitus (y/n)	−173.100	42.580			
Season, spring and summer vs. fall and winter	40.180	25.060			
Undiagnosed thyroid disease (euthyroid/hypothyroid/hyperthyroid)	16.520	37.650			
Depressive symptoms (CES-D), sum score	2.183	2.323			
Total intracranial volume, mm³	0.001	<0.001			

*BMI* body mass index, *CES-D* Center for Epidemiological Depression Scale, *HCV* hippocampal volume, *IPAQ* International Physical Activity Questionnaire, *MET* metabolic equivalent of task, *MRI* magnetic resonance imaging, *PSQI* Pittsburgh Sleep Quality Index, *SE* standard error.

Significant codes: *p < 0.017, **p < 0.01, ***p < 0.001.

Results further indicated that higher age and cardiovascular risk factors correlated with smaller hippocampal volumes, for example diabetes (right hemisphere, *β* (SE) = −139.5 (42.180), *p* < 0.001 and left hemisphere, *β* (SE) = −173.1 (42.58), *p* < 0.001), less physical activity (right hemisphere, *β* (SE) = −26.67 (12.68), *p* = 0.036) and higher alcohol consumption (right hemisphere, *β* (SE) = −1.945 (0.853), *p* = 0.023) (see SI). In further analysis of the interaction of sex and ghrelin levels on hippocampal volume, no sex differences were observed (left hippocampal volume *β*_ghrelin*sex_ (SE) = 96 (63.35), *p* = 0.13 and right hippocampal volume *β*_ghrelin*sex_ (SE) = 69.74 (69.07), *p* = 0.313).

## Discussion

In this well-characterized population-based study including a share of 5–7% participants with (non-medicated) depressive episode, analyses of BMI, self-reported depressive symptoms and peripheral ghrelin levels revealed a connection between higher BMI and more depressive symptoms, alongside lower fasting ghrelin levels. However, fasting ghrelin serum levels were not associated with the overall sum score of depressive symptoms in individuals living with obesity in our cohort. Exploratory analyses indicated that higher ghrelin levels were associated with decreased appetite and less self-reported weight loss within the last two weeks in individuals living with obesity. No significant associations emerged between ghrelin serum levels and hippocampal volumes.

The observed association between higher BMI and more depressive symptoms aligns well with previous research indicating a link between obesity and worse mental health^59,60^. While the effect size can be considered small (adjusted R-squared value of 0.026), suggesting that BMI explains only a small portion of the variance in depressive symptoms in our cohort, a linear association between higher weight status and depressive symptoms is particularly alarming from a public health perspective^61^, especially given the estimated rise of prevalence of obesity in the upcoming years^62^. However, alternative pathways linking obesity and depressive symptoms include reduced physical activity^63^ and adipose tissue-derived cytokines that influence serotonin metabolism^64^, representing important mechanisms that may contribute to depressive symptoms of BMI beyond those associated with ghrelin. It should also be noted that, although participants in the present study included individuals with a diagnosis of MDD, psychotropic medication was an exclusion criterion, which may bias the spectrum of severity of depressive symptoms in the sample.

Our finding indicating that higher BMI correlates with lower fasting ghrelin confirms the results of previous studies, showing that lower ghrelin relates to percentage body fat, general obesity^54^, and abdominal obesity in type 2 diabetes patients^65^. Furthermore, a comprehensive meta-analysis, incorporating 34 studies and comprising over 1863 participants, reinforced the clinical evidence indicating that individuals living with obesity exhibit lower baseline levels of circulating acylated ghrelin and desacylated ghrelin^7^. Interestingly, in this meta-analysis, subjects living with obesity displayed a shorter duration of acylated ghrelin suppression following meal intake, underscoring the significance of analyzing postprandial ghrelin levels in future research.

Against our hypothesis postulating a link between lower ghrelin and more frequent depressive symptoms, we did not find evidence for such an association in participants living with obesity, defined as a BMI ≥ 30 kg/m², in our cohort. This is consistent with a previous analysis including participants from all BMI strata (mean BMI of 26.99 ± 4.48) from the same cohort (*n* = 1092;^26^). Furthermore, others observed higher ghrelin in a moderate sample size of postmenopausal females (*n* = 55) with more severe compared to less severe depressive symptoms^66^.

These conflicting and somewhat surprising results also considering animal studies could relate to different factors. On the one hand, our sample size was relatively large and analyses were adjusted for a set of carefully selected confounders, and we specifically focused on participants living with obesity and related disturbances in ghrelin metabolism. The investigation focusing specifically on a BMI of 30 or greater was particularly relevant, as it provided insights into a more homogeneous group where the relationship between ghrelin and depressive symptoms might differ. For example, comorbidities associated with obesity, such as diabetes and undiagnosed thyroid disease, were notably more prevalent in the H3 sample, which was restricted to individuals with a BMI over 30 (see Table 1). This could speak for the robustness of our null findings compared to previous reports, implying a neglibigle or non-existent role of ghrelin in depressive symptoms in humans living with obesity. However, the etiology of depressive symptoms is considered complex and multifactorial^67^, rendering masking by a variety of still other factors possible. For example, perceived weight discrimination has been found to be a significant contributor to the association between depressive symptoms and obesity^68^. This emphasizes the importance of psychosocial variables in the relationship between obesity and depressive symptoms that should be accounted for as confounders in future research. Also, although the underlying mechanisms are not yet fully understood, studies emphasize the role of insulin resistance and related hyperinsulemia which may suppress ghrelin secretion, as ghrelin levels were lower in insulin-resistant adults with obesity compared to insulin-sensitive adults with obesity^69,70^. Further research exploring the connection between BMI, depressive symptoms and ghrelin levels should thus account for the potential influence of insulin resistance.

Interestingly, in our cohort of participants living with obesity, ghrelin levels were associated with subcategories of depressive symptom questions that related to decreased appetite and yet less weight loss within the last two weeks. This suggests that ghrelin levels could play a role in depressive symptoms related to appetite and weight regulation. The association between higher ghrelin levels and less weight loss is physiologically plausible, as ghrelin stimulates appetite and is implicated in energy balance mechanisms and weight gain. Seemingly contradictory at first glance, the association between higher ghrelin levels and decreased appetite could be explained as a metabolic adaptation to the loss of appetite as a depressive symptom by the release of hunger hormones. However, caution in interpreting these results is warranted as the results were obtained through exploratory analysis.

Further adding to the complexity of the involvement of ghrelin levels in depressive symptoms, animal studies suggest that a variety of different circumstances can affect whether ghrelin exerts antidepressant or depressiogenic effects. These conditions encompass distinctions between acute and chronic stress exposure^71,72^, the feeding status^73^, and whether animals had been under stress or were stress-free prior to ghrelin exposure^13^. This indicates that ghrelin’s effects on depressive symptoms may be highly context-dependent, influenced by both physiological and environmental factors. Future studies should consider examining the relationship between pre- vs. postprandial ghrelin levels and depressive symptoms, as well as the perceived stress levels of the participants.

Furthermore, to deepen our comprehension of the causal relationship between ghrelin levels and depressive symptoms, longitudinal studies and randomized controlled trials are needed. These methodologies would provide more robust evidence regarding temporality, causality, and effectiveness of possible interventions.

The null results regarding an association between ghrelin levels and hippocampal volume could imply that ghrelin levels do not affect brain structure, specifically hippocampal volume, in humans, somewhat contrasting with some animal studies that have reported neuroprotective effects of ghrelin^74,75^. However, hippocampal volume was measured cross-sectionally only. Therefore, trajectories of hippocampal volume over time as a better measure for hippocampal atrophy could offer further insights. Moreover, the impact of ghrelin on brain structure is likely complex, due to several interacting factors such as hormonal interactions, temporal dynamics and lifestyle factors influencing the association between ghrelin levels and hippocampal volume. While we controlled extensively for a set of carefully selected confounders, ghrelin interacts with various other hormones, such as leptin, insulin, and cortisol, which are also known to influence brain structure and function^61^. These interactions could modulate the effect of ghrelin on the hippocampus and therefore obscure the effect of ghrelin serum levels on hippocampal volume. Investigating these complex hormonal interactions could provide further insights into the potential indirect effects of ghrelin on brain structures.

We found that BMI and depressive symptoms were associated in females only in our cohort, confirming the results of previous research showing that the association in the overall sample is attributable to females^76^. This suggests that sex might also modify the relationship between BMI, ghrelin, and depressive symptoms. However, we could not observe sex differences in the associations between BMI and ghrelin levels, and neither between ghrelin levels and depressive symptoms in individuals with obesity, nor between ghrelin levels and hippocampal volume. However, note that sample sizes were substantially smaller for these analyses, rendering limited power to detect effects possible. Future studies with larger samples sizes and additional measures of estrogen levels of participants in connection with ghrelin and depressive symptoms are warranted, as estrogen may enhance antidepressant-like effects of ghrelin^31^. In addition, factors related to gender (e.g., gender-specific discrimination) could have contributed to the observed stronger association between obesity and depression in females.

One limitation of this study is the pragmatic pre-analytical treatment of the blood samples used for the ghrelin measurement, preventing the possibility to distinguish both ghrelin isoforms (acyl-ghrelin and desacyl-ghrelin) in the analysis. Measuring both acyl-ghrelin and desacyl-ghrelin and not only total ghrelin provides a more accurate representation, as desacyl-ghrelin is supposed to be inactive^10^. This methodological limitation of our study may have contributed to the lack of observed effects of ghrelin serum levels on depressive symptoms in indivudals with obesity, implicating for further research to differentiate between ghrelin isoforms in order to more accurately assess their potential role in the interplay between obesity and depressive symptoms. Note that our sample, similar to other epidemiological data^77^, included only a small proportion (5–7%) of participants showing signs of clinically relevant depressive symptoms, rendering limited power to detect associations in the sub-clinical range possible. Additionally, it is important to emphasize that the cross-sectional design of this study renders causal inferences from our results impossible. Another critical limitation of this study is the reliance on BMI as the sole metric for obesity, as it does not account for body fat distribution – a key determinant of metabolic health^78^. Furthermore, the existing body of literature on peripheral biomarkers in mental disorders exhibits systematic bias, as highlighted in a recent umbrella review that identifies widespread methodological heterogeneity and underpowered studies in this field, which limits the generalizability of findings^79^. While this represents a critical methodological concern, our study addresses this limitation through a large, well-characterized population-based sample that enhances the reliability of our results.

Strengths of this study are the population-based sample offering better potential for generalizability, with a large sample size, advanced 3T MRI for hippocampus volumetry, and the use of validated questionnaires. Nonetheless, a valid diagnosis of MDD can only be made by a health care professional through a structured clinical interview. Therefore, it was not possible to distinguish between the subtypes of MDD, e.g., atypical depression. As different subtypes of MDD exhibit diverse patterns of appetite and eating behavior^80^, exploring these subtypes could be insightful when examining the association between ghrelin levels and depressive symptoms in participants living with obesity. Accordingly, our study focused on depressive symptoms as a transdiagnostic observation rather than on the specific diagnosis of MDD.

This study examined the relationships between BMI, depressive symptoms, and ghrelin levels in a population-based cohort of individuals. Our findings revealed that higher BMI is associated with more depressive symptoms and lower fasting ghrelin levels. Yet no association was found between fasting ghrelin levels and the overall severity of depressive symptoms in participants living with obesity. These results underscore the need for future research to consider pre- and postprandial ghrelin levels and its interaction with other hormones crucial to metabolic homeostasis such as insulin and leptin, alongside the analysis of structured clinical interviews for MDD. While ghrelin did not mediate the relationship between BMI and depressive symptoms in individuals with obesity in our study and thus cannot serve as a biomarker for depressive symptoms-obesity comorbidity, our results underscore the critical need to prioritize mental health support for women with obesity, as they exhibited a stronger association between BMI and depressive symptoms compared to men, highlighting sex-specific susceptibility in obesity-related psychological distress. These findings advocate for clinical and societal interventions that address systemic and biological factors disproportionately affecting women’s mental health in obesity, e.g., weight discrimination. Ultimately, our study emphasizes the multi-factorial nature of the association between depressive symptoms and obesity, calling for comprehensive approaches in future investigations to unravel these complex interrelationships. Particularly longitudinal and interventional studies are promising approaches to better understand the complex interplay between metabolic and psychological factors.

## Supplementary information

Supplementary Information

## Data Availability

Access to the data used in this study was granted by LIFE (Leipziger Forschungszentrum für Zivilisationserkrankungen) under project agreement PV-695. The data are managed and distributed by LIFE (https://www.uniklinikum-leipzig.de/einrichtungen/life) in accordance with individual project proposals and the data protection regulations of Leipzig University.

## Code availability

All code used for analysis and visualization in this study is publicly available via GitLab: https://gitlab.gwdg.de/omega-lab/obesity_depression_ghrelin. The repository contains the scripts and documentation necessary to reproduce the results presented in the manuscript. For further details or questions regarding the code, please contact the corresponding author.
