# Author Correction: Fasting ghrelin as mediator between obesity and depressive symptoms: a pre-registered study

**DOI:** 10.1038/s44184-026-00225-2

**Published:** 2026-07-06

**Authors:** Konrad Jakob Endres, Laurenz Lammer, Frauke Beyer, Daria E. A. Jensen, Dirk Alexander Wittekind, Margrit Löbner, Arno Villringer, Julia Sacher, A. Veronica Witte

**Affiliations:** 1 Department of Neurology, Max Planck Institute for Human Cognitive and Brain Sciences, Leipzig, Germany; 2 Cognitive Neurology, University of Leipzig Medical Center, Leipzig, Germany; 3 Bordeaux Population Health Research Center, Inserm, University of Bordeaux, Bordeaux, France; 4 LeiCeM - Leipzig Center of Metabolism, Leipzig University, Leipzig, Germany; 5 Institute of Laboratory Medicine, Clinical Chemistry and Molecular Diagnostics, Leipzig University, Leipzig, Germany; 6 Department of Psychiatry and Psychotherapy, Leipzig University, Leipzig, Germany; 7 Institute of Social Medicine, Occupational Health and Public Health, Faculty of Medicine, Leipzig University, Leipzig, Germany; 8 Center for Integrative Female Health & Gender Medicine, University of Leipzig Medical Center, Leipzig, Germany; 9 Medical Department III – Endocrinology, Nephrology, Rheumatology, University Leipzig, Leipzig, Germany; 10 German Center for Child and Adolescent Health (DZKJ), partner site Leipzig/Dresden, Greifswald, Germany

**Keywords:** Obesity, Neuroscience, Biomarkers, Epidemiology, Translational research

Correction to: *npj Mental Health Research* 10.1038/s44184-026-00217-2, published online 19 May 2026

In this article, the author names ‘Daria E.A. Jensen’ and ‘A. Veronica Witte’ were incorrectly written as ‘Daria Jensen’ and ‘Veronica Witte’ respectively. The original article has been corrected.

