# Supplementary material for: Fasting ghrelin as mediator between obesity and depressive symptoms: a pre-registered study
Source: Npj Ment Health Res. 2026 May 19;5:28. doi: 10.1038/s44184-026-00217-2 (PMC13187281; doi:10.1038/s44184-026-00217-2)
Supplement: Supplementary file 1 — Supplementary Information [file 44184_2026_217_MOESM1_ESM.pdf]

Supplementary table 1: *Results from multiple linear regression analyses examining the relationship between log-transformed ghrelin levels and specific physical depressive symptoms (IDS-SR subitems)*

	**β**	**SE**	**t-value**	**p-value**	**significance**
**Log-transformed Ghrelin levels in sample for H3 (n = 263)**<br>**adjusted R-squared = 0.266 F-statistic = 3.23 on 7 and 87 DF, p-value = 0.004**					
IDS_SUM_sleep	0.002	0.018	0.102	0.919	
**IDS_I11 (decreased appetite)**	**0.273**	**0.088**	**3.105**	**0.003**	**
IDS_I12 (increased appetite)	0.354	0.194	1.823	0.072	
**IDS_I13 (weight decrease)**	**-0.111**	**0.043**	**-2.568**	**0.012**	*
IDS_I14 (weight increase)	-0.051	0.046	-1.123	0.265	
IDS_I28 (gastrointestinal symptoms)	-0.076	0.045	-1.703	0.092	
IDS_SUM_motor	0.008	0.030	0.271	0.787	
Abbreviations: **DF**: Degrees of Freedom, **IDS-SR**: Inventory of Depressive Symptoms – Self Rating, **SE**: Standard Error					
